# MR-Spectroscopy of GABA and Glutamate/Glutamine Concentrations in Auditory Cortex in Clinical High-Risk for Psychosis Individuals

**DOI:** 10.3389/fpsyt.2022.859322

**Published:** 2022-03-29

**Authors:** Tineke Grent-’t-Jong, Ruchika Gajwani, Joachim Gross, Andrew I. Gumley, Stephen M. Lawrie, Matthias Schwannauer, Frauke Schultze-Lutter, Stephen R. Williams, Peter J. Uhlhaas

**Affiliations:** ^1^Institute of Neuroscience and Psychology, University of Glasgow, Glasgow, United Kingdom; ^2^Department of Child and Adolescent Psychiatry, Charité – Universitätsmedizin Berlin, Berlin, Germany; ^3^Mental Health and Wellbeing, Institute of Health and Wellbeing, University of Glasgow, Glasgow, United Kingdom; ^4^Institute for Biomagnetism and Biosignalanalysis, University of Münster, Münster, Germany; ^5^Department of Psychiatry, University of Edinburgh, Edinburgh, United Kingdom; ^6^Department of Clinical Psychology, University of Edinburgh, Edinburgh, United Kingdom; ^7^Department of Psychiatry and Psychotherapy, Medical Faculty, Heinrich Heine University, Düsseldorf, Germany; ^8^Department of Psychology, Faculty of Psychology, Airlangga University, Surabaya, Indonesia; ^9^University Hospital of Child and Adolescent Psychiatry and Psychotherapy, University of Bern, Bern, Switzerland; ^10^Division of Informatics, Imaging and Data Science, Faculty of Biology, Medicine and Health, University of Manchester, Manchester, United Kingdom

**Keywords:** clinical high-risk, psychosis, E/I balance, GABA, glutamate, Glx, MR-spectroscopic imaging

## Abstract

Psychosis involves changes in GABAergic and glutamatergic neurotransmission in auditory cortex that could be important for understanding sensory deficits and symptoms of psychosis. However, it is currently unclear whether such deficits are present in participants at clinical high-risk for psychosis (CHR-P) and whether they are associated with clinical outcomes. Magnetic Resonance Spectroscopy (MEGAPRESS, 1H-MRS at 3 Tesla) was used to estimate GABA, glutamate, and glutamate-plus-glutamine (Glx) levels in auditory cortex in a large sample of CHR-P (*n* = 99), CHR-N (clinical high-risk negative, *n* = 32), and 45 healthy controls. Examined were group differences in metabolite concentrations as well as relationships with clinical symptoms, general cognition, and 1-year follow-up clinical and general functioning in the CHR-P group. Results showed a marginal (*p* = 0.039) main group effect only for Glx, but not for GABA and glutamate concentrations, and only in left, not right, auditory cortex. This effect did not survive multiple comparison correction, however. Exploratory *post-hoc* tests revealed that there were significantly lower Glx levels (*p* = 0.029, uncorrected) in the CHR-P compared to the CHR-N group, but not relative to healthy controls (*p* = 0.058, uncorrected). Glx levels correlated with the severity of perceptual abnormalities and disorganized speech scores. However, in the CHR-P group, Glx levels did not predict clinical or functional outcomes. Accordingly, the findings from the present study suggest that MRS-measured GABA, glutamate and Glx levels in auditory cortex of CHR-P individuals are largely intact.

## Introduction

Schizophrenia (ScZ) is a severe mental illness associated with pronounced cognitive and sensory impairments that are thought to emerge from underlying impairments of neural circuits (1, 2). These deficits in sensory and cognitive operations correlate with clinical (3, 4) as well as functional outcomes (5, 6) and are not improved by current pharmacological treatment options (7), so identifying the neurobiological origin of such deficits is a priority of current research.

Recent evidence has implicated a disturbance in the balance between excitation and inhibition (E/I-balance), a mechanism that assures efficient information transfer in neural networks (8, 9), as a potential target for understanding cognitive deficits in ScZ (10). During normal brain functioning, E/I-balance emerges from the interplay between inhibitory GABAergic interneurons and excitatory (e.g., glutamatergic) synapses (11). A shift toward increased excitation could result in elevated basal noise levels and lower signal-to-noise ratio (12). On the other hand, a shift toward increased inhibition may result in lower signal-strength, resulting in an impaired signal-propagation of sensory inputs (13).

Evidence for E/I-balance abnormalities in ScZ comes from post-mortem studies that have examined alterations in GABAergic interneuron properties as well as N-methyl-D-aspartate receptor (NMDA-R) density. Specifically, reductions in the activity level of Parvalbumin-expressing (PV+) but also of Somatostatin-expressing (ST) interneurons have been identified in prefrontal cortex that also extend into sensory areas such as auditory cortex (14) and subcortical regions (15, 16). Furthermore, a reduction in the NR1 subunit (17), but also in other NMDA-R subunits, such as the NR2C subunit (18–20), have been found, primarily in dorsolateral prefrontal cortex.

Further evidence for E/I-balance alterations come from studies using Magnetic Resonance Spectroscopy (MRS) to investigate regional levels of GABA glutamate and/or Glx (glutamate-plus-glutamine). Recent meta-analyses have suggested regional as well as stage-specific differences in levels of glutamate and/or Glx (21, 22). For example, in ScZ patients, elevated levels of glutamate and Glx are most consistently found in limbic/subcortical structures, such as the basal ganglia, thalamus, and medial temporal lobe, whereas decreased levels are reported for medial prefrontal areas (21–23).

As ScZ can be preceded by a prodromal period of up to 5 years during which attenuated psychotic symptoms (APS) as well as behavioral and cognitive changes already exist (24, 25), identifying individuals at clinical high risk for developing psychosis (CHR-P) is important, as it enables the opportunity to develop appropriate strategies for risk prediction and early intervention (26, 27). Recent MRS studies in CHR-P individuals so far have mostly documented a decrease in hippocampal and thalamic glutamate levels (28, 29). Furthermore, increases in both GABA and glutamate have also been reported in associative-striatal and medial prefrontal regions in CHR-P participants (30, 31), with the highest striatal glutamate levels found in those who later transitioned to psychosis (32).

Few MRS studies, however, have investigated sensory areas, such as auditory regions, despite the fact that auditory cortex functioning is of crucial importance for understanding circuit dysfunctions in ScZ, given the profound disruptions in auditory processing that involve impaired perception of stimulus features (33) as well as deviance detection (34, 35). Moreover, abnormal auditory processing is also present already in CHR-P participants (36), and pronounced in those with more severe psychopathology and/or who transition to psychosis (37–39). MRS-studies in ScZ patients have reported increased GABA levels in right auditory regions (40), as well as lower levels of glutamate (41) and Glx (42) in the left superior temporal cortex. However, it is currently unclear whether such changes are also present in CHR-P populations.

In the current study, we therefore examined GABA, glutamate and Glx levels in right and left auditory cortex, in a sample of 99 anti-psychotic medication naïve CHR-P participants, 32 participants with affective disorders and substance abuse disorders (CHR-N), and 45 healthy controls (HC).

Moreover, we established whether MRS-measures would be related to clinical outcomes in the CHR-P group, such as persistence of APS and functional outcomes.

We predicted that CHR-P participants would be characterized by reduced levels of glutamate and/or Glx in left auditory regions and increased levels of GABA in right auditory regions, consistent with the earlier findings in ScZ patients (42). In addition, we hypothesized that those changes would be most pronounced for individual with greater symptom severity and/or poor clinical outcomes.

## Materials and Methods

Data were collected as part of the YouR-Study (The Youth Mental Health Risk and Resilience Study), which is a longitudinal study funded by the Medical Research Council (MRC) (43) that aims at identifying neurobiological mechanisms and predictors of emerging psychosis. Baseline data collection was conducted between March 2015 and November 2019. The study was approved by the ethical committees of the NHS Research Ethical Committee Glasgow & Greater Clyde. All participants provided written informed consent.

### Participants

Auditory cortex 1H-MRS data was included from three groups of participants: (1) participants meeting CHR-P criteria; (2) participants who did not meet CHR-P criteria but met criteria for non-psychotic disorders, such as mood and anxiety disorders, eating disorders and substance abuse (CHR-N), indicated by the Mini-International Neuropsychiatric Interview (44); and (3) healthy control individuals (HC) without an axis I diagnosis or family history of psychotic disorders. CHR-P status was confirmed by meeting ultra-high-risk (UHR) criteria according to the Comprehensive Assessment of At-Risk Mental States (CAARMS) interview (45) and the Cognitive Disturbances and Cognitive-Perceptive Basic Symptoms criteria according to the Schizophrenia Proneness Instrument, Adult version [SPI-A: (46)]. In total, 25 CHR-Ps met only basic symptom criteria, *n* = 23 met only UHR, and 51 CHR-Ps met both basic symptom and UHR criteria. Individuals classified as CHR-N did not meet threshold for UHR and basic symptoms, but met criteria for other non-psychotic disorders, excluding autism, ADHD, or first-degree relatives with ScZ. Cognition was assessed with the Brief Assessment of Cognition in Schizophrenia [BACS: (47)].

### Clinical Follow-Up

Participants meeting CHR-P criteria were reassessed at 3-, 6-, 9-, 12-, 18-, 24-, 30-, and 36-month intervals to examine persistence of APS and functional outcomes, using with the CAARMS interview.

### 1H-MRS Data Acquisition

**FIGURE 1:**
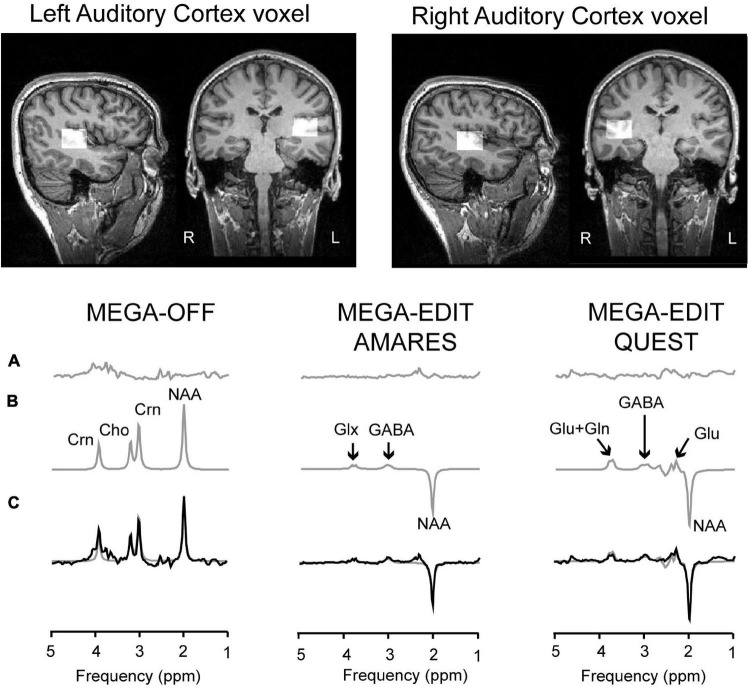
Voxel placement and MRS analyses pipeline. Top two panels show an example of the placement of the voxels in right and left auditory cortex. The bottom-line figures show example spectra and model fits. More specifically, the bottom traces **(C)** show acquired data from three different subjects, with the model fit overlaid as a smooth gray line. The model fits are separately shown in panel **(B)**, with the residuals shown in the top traces **(A)**. The left-hand panel shows data from the control sub-spectrum, in which the MEGA editing pulse is set away from the GABA C3 frequency. The right-hand panels show edited spectra (subtraction of MEGA-OFF from MEGA-ON) fitted either by AMARES (middle) or QUEST/QUASAR (right). Peaks are labeled as Cho–choline–containing compounds, Crn–total creatine, GABA, glutamate–glu, glutamate plus glutamine–Glx, and NAA–N-Acetyl-Aspartate.

Data from bilateral auditory cortex were acquired on a Siemens 3T-Tim-Trio scanner, using a 32-channel head coil for reception. First, T1-weighted anatomical MRI images were collected using 3D-MPRAGE sequences (192 slices, voxel size 1 mm^3^, FOV = 256 × 256 × 176 mm, *TR* = 2,250 ms, *TE* = 2.6 ms, *FA* = 9°), and resliced into axial and coronal views to allow more precise and consistent placement of the MRS voxels of interest. Voxels of 2 cm^3^ were then placed in right (RAUD) or left auditory cortex (LAUD), positioned over Heschl’s gyrus, horizontally aligned with the lower bank of the supra-temporal plane on the coronal slide (Figure 1, top panel), and optimized in location to a final position with minimal Cerebral Spinal Fluid (CSF) contamination. FASTMAP (48) semi-automatic shimming of the voxel was used to improve local field homogeneity in the area of interest. A total of three scans were acquired, including a full spectrum acquisition, a GABA-edited MEGA-PRESS (WIP: VB-17A) scan (128 trials), and an unsuppressed water scan to provide an absolute concentration reference (64 trials). MEGA-PRESS scanning parameters included: TR/TE 1,500/68 ms, 1.9 ppm ON- and 1.5 ppm OFF-resonance editing pulse frequencies (symmetric editing to suppress macromolecule contribution), 44 Hz editing Gaussian pulse bandwidth, 50 Hz water suppression, 90^°^ flip angle, acquisition bandwidth of 1,200 Hz, duration 426 ms, number of points 512.

The number of available samples differed for LAUD and RAUD voxels. In total across the three groups, 135 participants had recordings from both voxels, 62 only from right, and 1 only from left auditory cortex. As a result, analyses were run separately for both voxels. After removal of participants with poor quality data, the final sample included 92 CHR-P, 30 CHR-N, and 44 HC subjects for the RAUD voxel, and 60 CHR-P, 20 CHR-N, and 31 HC subjects for the LAUD voxel.

Cleaning involved removing data with incomplete/missing metabolite concentrations, and/or data that were more than 2 standard deviations away from the mean across groups (within each voxel) for linewidth of N-acetyl-aspartate [NAA] and/or unsuppressed water, and/or absolute Cramér-Rao Lower Bound (CRLB) values. Absolute CRLB values were favored over percentages following most recent recommendations for clinical situations of potential low concentrations of metabolites (49, 50). A full overview of data quality measures is presented in Table 1.

**TABLE 1:** Metabolite concentrations, voxel segmentation fractions, and data quality measures.

	Left auditory voxel mean (SD)			Right auditory voxel mean (SD)		
	HC (*n* = 31)	CHR-N (*n* = 20)	CHR-P (*n* = 60)	HC (*n* = 44)	CHR-N (*n* = 30)	CHR-P (*n* = 92)
**Metabolite concentration**						
GABA	1.630 (0.27)	1.717 (0.35)	1.624 (0.29)	1.674 (0.28)	1.632 (0.24)	1.644 (0.30)
Glx	5.155 (0.71)	5.224 (0.55)	4.865 (0.77)	5.234 (0.80)	5.268 (0.78)	5.293 (0.92)
Glu	1.069 (0.15)	1.161 (0.25)	1.074 (0.17)	1.045 (0.14)	1.045 (0.13)	1.017 (0.14)
Glx-GABA ratio	3.23 (0.63)	3.13 (0.60)	3.09 (0.79)	3.21 (0.68)	3.31 (0.81)	3.34 (0.94)
Glu-GABA ratio	0.67 (0.11)	0.68 (0.11)	0.68 (0.15)	0.63 (0.09)	0.65 (0.08)	0.64 (0.14)
**Voxel segmentation**						
GM fraction	0.509 (0.06)	0.515 (0.05)	0.504 (0.08)	0.525 (0.06)	0.500 (0.07)	0.498 (0.07)
WM fraction	0.449 (0.07)	0.440 (0.07)	0.450 (0.09)	0.433 (0.07)	0.461 (0.08)	0.459 (0.09)
CSF fraction	0.042 (0.02)	0.045 (0.02)	0.046 (0.02)	0.042 (0.02)	0.039 (0.02)	0.043 (0.03)
GM/WM ratio	1.18 (0.33)	1.21 (0.30)	1.20 (0.40)	1.27 (0.34)	1.15 (0.36)	1.16 (0.39)
**Data quality**						
NAA/Water	24.4 (2.0)	25.7 (5.7)	24.6 (2.0)	24.4 (2.2)	23.9 (3.3)	24.1 (2.4)
Creatine/Water	16.5 (1.1)	17.2 (3.7)	16.6 (1.3)	16.7 (1.5)	16.7 (1.4)	16.6 (1.4)
NAA/Creatine	1.5 (0.1)	1.5 (0.1)	1.5 (0.1)	1.5 (0.1)	1.4 (0.2)	1.5 (0.1)
NAA line width	6.3 (1.0)	5.9 (1.0)	5.8 (0.9)	6.1 (0.9)	6.2 (1.3)	6.2 (1.1)
Water line width	7.6 (0.8)	7.2 (0.7)	7.4 (0.8)	7.4 (0.9)	7.3 (0.9)	7.4 (0.8)
**CRLB absolute values: AMARES**						
NAA	0.94 (0.17)	0.93 (0.19)	0.89 (0.18)	0.94 (0.22)	0.94 (0.25)	0.92 (0.19)
GABA	1.09 (0.20)	1.08 (0.19)	1.11 (0.22)	1.12 (0.23)	1.07 (0.27)	1.06 (0.22)
Glx	0.81 (0.12)	0.81 (0.14)	0.78 (0.14)	0.81 (0.17)	0.81 (0.18)	0.80 (0.14)
**% CRLB: AMARES**						
NAA	2.3 (0.5)	2.2 (0.5)	2.2 (0.6)	2.0 (0.5)	2.0 (0.4)	2.0 (0.4)
GABA	20.3 (3.8)	20.0 (4.0)	21.8 (6.8)	17.9 (4.0)	17.8 (4.4)	17.7 (4.0)
Glx	12.0 (2.5)	12.3 (3.7)	12.9 (3.8)	10.6 (3.2)	10.5 (2.2)	10.4 (2.4)
**CRLB absolute values: QUEST**						
NAA	0.13 (0.02)	0.13 (0.03)	0.12 (0.03)	0.13 (0.03)	0.13 (0.03)	0.13 (0.02)
Glu	0.13 (0.02)	0.13 (0.02)	0.13 (0.03)	0.13 (0.02)	0.13 (0.02)	0.12 (0.02)
**% CRLB: QUEST**						
NAA	2.1 (0.5)	2.0 (0.6)	2.1 (0.7)	1.9 (0.5)	1.9 (0.5)	2.0 (0.5)
Glu	7.4 (1.9)	7.4 (1.8)	7.8 (2.6)	6.6 (1.6)	6.6 (1.4)	6.7 (1.7)

*HC, healthy control group; CHR-N, Clinical-High-Risk negative group; CHR-P, Clinical-High-Risk positive group; NAA, N-Acetyl-Aspartate; GABA, gamma Aminobutyric Acid; Glx, Glutamate-plus-Glutamine; Glu, Glutamate; CRLB, Cramér-Rao Lower Bound; GM, gray matter; WM, white matter; CSF, cerebral spinal fluid; AMARES and QUEST, non-linear-least-squares and time-domain quantification algorithm, respectively (implemented in JMRUI MR-Spectroscopy analysis software); SD, standard deviation of the mean; metabolite concentrations referenced to water.*

### Post-processing of MRS Data

MEGA-OFF, MEGA-ON, and unsuppressed water spectra were processed in jMRUI v6.0 (51). Firstly, edited spectra were generated by subtracting MEGA-OFF from MEGA-ON and GABA, Glx and MEGA edited-NAA were quantified using the AMARES routine (52). Based on previous work (53), we used QUEST/QUASAR (54) to estimate glutamate and glutamine separately from edited MEGA-PRESS spectra (see Figure 1 for AMARES and QUEST example spectra). This approach has been shown to estimate levels of glutamate and glutamine that are consistent with literature values, provided spectroscopic quality is good, with the principal determinant being the linewidth of NAA (<10.6 Hz: means were around 6 Hz, see Table 1). As only glutamate spectral quality measures were met (%CRLB for LAUD 8.2 ± 3.1%, RAUD 7.1 ± 3.0%), but not those for glutamine (%CRLB for LAUD 40.9 ± 41.1%, RAUD 18.2 ± 26.7%), only glutamate levels are reported.

The edited MEGA-PRESS ON spectra were fitted to a basis set consisting of NAA, GABA, and glutamate in which the full edited spectra were represented. The basic model sets were generated from a simulated MEGA-PRESS sequence using the routine NMRSCOPE-B. GABA was fitted as a single Gaussian line with the linewidth constrained to be between 20 and 25 Hz at full width, half maximum height with the frequency constrained to be +1.00 ppm away from NAA. Glx was fitted as a symmetric doublet centered at 3.76 ppm with a Lorentzian line shape, a 10.4 Hz separation, with the same linewidth as NAA. NAA was fitted as a Lorentzian line, 180° out of phase to GABA and Glx. The MEGA-OFF spectra were also fitted by AMARES to give estimates of the unedited NAA (peak at 2.02 ppm), of total creatine (Crn: peak at 3.03 ppm) and of choline-containing compounds (Cho: peak at 3.21 ppm; glycerol-phosphorylcholine, phosphorylcholine and free choline).

Finally, data was referenced to the separately collected unsuppressed water concentration and corrected for contamination of cerebrospinal fluid (CSF) fractions (metabolite concentration * 1/1-CSF fraction).

### Statistical Analysis of Demographic, Clinical, and Cognitive Data

Group differences in demographic data, clinical assessment scores, as well as cognition (BACS scores) were assessed with non-parametric Kruskal-Wallis tests, using an alpha-level of 0.05 (2-sided tests). *Post-hoc* comparisons were Games-Howell corrected for multiple comparisons and corrected for ties. BACS data were first z-normalized to HC data, separately for females and males. Gender differences were tested with Chi-square tests.

### Statistical Analysis of MRS Data

As the number of available data were different for the RAUD and LAUD voxels, the main analyses described below were run for each voxel separately, using an alpha-level of 0.05 (2-sided).

First, potential group differences in voxel segmentation, NAA and Creatine levels were investigated using one-way Welch’s ANOVAs with the dependent-variables NAA/water, Creatine/water, gray matter (GM), white matter (WM), cerebrospinal fluid (CSF), and GM/WM ratio, and the fixed-factor GROUP (HC, CHR-N, CHR-P). Both NAA and Creatine concentrations were obtained from the MEGA-OFF sequence, while water concentration was estimated from the unsuppressed water scan.

Subsequent analyses included General Linear Model ANCOVAs, using the dependent-variables GABA, Glx, glutamate (Glu), Glx/GABA ratio, and Glu/GABA ratio, and the fixed-factor GROUP (HC, CHR-N, and CHR-P), with AGE, GENDER and the ratio between GM and WM as covariates. Subsequent *post-hoc* pairwise comparisons for significant metabolites were Games-Howell corrected to control for multiple comparisons.

In addition, *post-hoc* analysis included two sets of follow-up CHR-P subgroups: 1) APS-persistent vs. APS non-persistent group, based on APS-criteria both at baseline and during 1-year follow-up, and 2) CHR-P participants with either good or poor functional outcomes (GAF scores good ≥ 65, poor < 65) at their latest follow-up assessment.

Finally, correlations between significant LAUD Glx concentrations and clinical, social and cognitive functioning, as well as age, known to correlate with Glx values (22, 55), were tested across the CHR-N and CHR-P groups, using linear regression with backward selection to identify the strongest predictors of abnormal metabolite concentrations. Potential collinearity issues were checked in the final model with VIF statistics.

## Results

### Demographic, Clinical, and Cognitive Data

There were no differences in age, or gender distribution between groups (Table 2). CHR-P and CHR-N groups, however, differed from HC in GAF scores (CHR-N: *p* < 0.001; CHR-P: *p* < 0.001) as well as in GF-social functioning (CHR-N: *p* = 0.002; CHR-P: *p* < 0.001), but GF-role was different from HC only in the CHR-P group (*p* < 0.001). Furthermore, the CHR-P group had less years of education, compared to controls (*p* = 0.017), and general cognition was affected only in CHR-Ps, who performed worse than controls on the BACS Token Motor task (*p* < 0.001), Symbol Coding task (*p* = 0.046), and had lower BACS composite scores (*p* = 0.018).

**TABLE 2:** Demographic, general cognition, and clinical assessment data.

	HC	CHR-N	CHR-P	Statistical results^a^
**Number of participants**	45	32	99	
**Age:** years, mean (SD)	22.7 (3.6)	22.3 (4.5)	21.7 (4.4)	No group differences
**Gender:** male/female (% male)	16/29 (35.5)	7/25 (21.2)	27/72 (27.2)	No group differences
**Education:** years (SD)	16.6 (2.8)	16.2 (3.2)	15.1 (3.1)	Group: H(2) = 9.8, *p* = 0.007 CHR-P < HC: *p* = 0.017
**BACS^b^**: mean (SD)				
Verbal memory	51 (9.3)	0.02 (1.2)	−0.32 (1.2)	No group differences
Digit sequencing	21 (2.8)	0.19 (1.1)	−0.29 (1.6)	No group differences
Token motor	80 (11.6)	−0.66 (1.1)	−0.87 (1.3)	Group: H(2) = 12.1, *p* = 0.002 CHR-P < HC: *p* < 0.001
Verbal fluency	59 (13.8)	−0.12 (1.0)	−0.07 (1.2)	No group differences
Symbol coding	72 (11.3)	0.03 (1.4)	−0.51 (1.2)	Group: H(2) = 9.9, *p* = 0.007 CHR-P < HC: *p* = 0.046
Tower of London	19 (1.8)	0.04 (1.3)	−0.21 (1.5)	No group differences
Total/Composite score	302 (25.2)	−0.13 (1.2)	−0.61 (1.4)	Group: H(2) = 7.3, *p* = 0.025 CHR-P < HC: *p* = 0.018
**CAARMS** **scores:** mean (SD)				
Unusual Thought Content (UTC)	0.0 (0.1)	0.8 (1.5)	5.9 (7.7)	−
Non-Bizarre Ideas (NBI)	0.1 (0.9)	2.6 (3.5)	10.6 (8.6)	−
Perceptual Abnormalities (PA)	0.3 (1.2)	1.8 (2.4)	8.7 (6.3)	–
Disorganized Speech (DS)	0.0 (0.3)	1.6 (2.8)	5.2 (5.4)	–
Total severity	0.5 (2.0)	6.8 (6.2)	30.4 (17.6)	Group: H(2) = 113.8, *p* < 0.001 CHR-P < HC: *p* < 0.001 CHR-N < HC: *p* < 0.001 CHR-P < CHR-N: *p* < 0.001
**SPI-A^c^ scores:** mean (SD)				
COPER items only	0	0	34	–
Total score, mean (SD)	0.2 (0.5)	0.6 (1.1)	8.2 (8.8)	
COGDIS items only	0	0	11	–
Total score, mean (SD)	0.1 (0.3)	1.4 (3.2)	6.3 (6.2)	
Both COGDIS and COPER items	0	0	30	–
Neither COGDIS nor COPER items	45	34	24	–
Total severity	0.2 (0.5)	1.2 (1.9)	10.6 (10.5)	Group: H(2) = 99.4, *p* < 0.001 CHR-P > HC: *p* < 0.001 CHR-N > HC: *p* = 0.022 CHR-P > CHR-N: *p* < 0.001
**GAF:** mean (SD)	87.8 (5.8)	69.4 (13.0)	57.1 (13.3)	Group: H(2) = 97.3, *p* < 0.001 CHR-P < HC: *p* < 0.001 CHR-N < HC: *p* < 0.001 CHR-P < CHR-N: *p* < 0.001
**GF-role**: mean (SD)	8.6 (0.7)	8.3 (0.7)	7.4 (1.2)	Group: H(2) = 49.1, *p* < 0.001 CHR-P < HC: *p* < 0.001 CHR-P < CHR-N: *p* < 0.001
**GF-social**: mean (SD)	8.8 (0.4)	8.3 (0.7)	7.5 (1.2)	Group: H(2) = 56.0, *p* < 0.001 CHR-P < HC: *p* < 0.001 CHR-N < HC: *p* = 0.002 CHR-P < CHR-N: *p* < 0.001

*HC, healthy control participants; CHR-N, Clinical High Risk for non-psychotic disorders; CHR-P, clinical high risk for psychotic disorders; BACS, Brief Assessment of Cognition in Schizophrenia; CAARMS, Comprehensive Assessment of At Risk Mental States; SPI-A, Schizophrenia Proneness Instrument, Adult version; COGDIS, Cognitive Disturbances criterion; COPER, Cognitive-Perceptive Basic Symptoms criterion; GAF, global assessment of functioning; GF, global functioning; SD, standard deviation of the mean.*

*^a^Except for “gender” statistical testing, which are based on Chi-Square tests, all other tests are based on non-parametric Kruskal-Wallis H-tests: alpha = 0.05, 2-sided, adjusted for ties, post-hoc Games-Howell corrected for multiple comparisons.*

*^b^BACS scores for clinical groups are standardized to control group data, controlled for gender.*

*^c^Threshold COPER/COGDIS is number of items with a score of 3 or more.*

### MRS Data: Quality Control

After removing data with partially missing metabolite concentrations or violations of the other quality control measures (Line width NAA and water, and CRLB values), group means and standard deviations for NAA and Creatine concentration, as well as GM, WM, CSF, or GM/WM ratios were not different between groups (HC, CHR-N, and CHR-P), neither for the LAUD nor for the RAUD voxel (Table 1).

### MRS Data: Main Group Analyses

**FIGURE 2:**
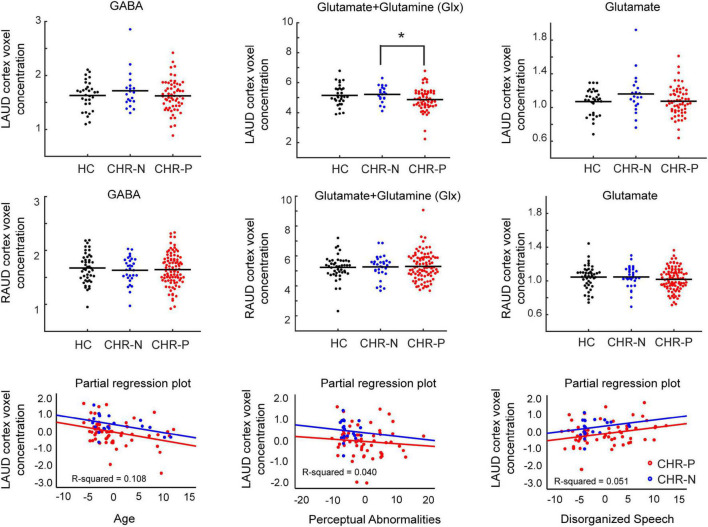
Metabolite concentrations and correlations. Distributions of main metabolite concentrations (GABA, Glx, and Glutamate), referenced to water, shown for left (LAUD: top row) and right (RAUD: second row) cortex voxel, and for all main groups (HC, CHR-N, and CHR-P). Significant group differences are indicated with an asterisk. Group means are indicated by a horizontal line. The bottom row shows scatter plots of partial regression correlations between LAUD z scored Glx levels and age of the participants (left), CAARMS Perceptual Abnormality scores (middle), and CAARMS Disorganized Speech scores (right). Groups are color coded, with black used for the HC group, blue for the CHR-N group and red for the CHR-P group. HC, healthy controls; CHR-N, Clinical-High-Risk negative: CHR-P, Clinical-High-Risk positive group; LAUD, left auditory MRS voxel; RAUD, right auditory MRS voxel.

ANCOVA analyses revealed no GROUP effects in any metabolites in the RAUD voxel (all p-values > 0.34). In contrast, the LAUD voxel showed a significant GROUP difference in Glx [*F*(2, 107) = 3.3, *p* = 0.039, uncorrected, partial η^2^ = 0.060] that was affected by the AGE [*F*(1, 107) = 10.3, *p* = 0.002, partial η^2^ = 0.089], but not by GENDER or GM-WM ratio. Exploratory *post-hoc* tests, including only AGE as covariate, revealed that this was driven mostly by the difference between the CHR-N and CHR-P groups (*p* = 0.029 uncorrected, *p* = 0.072 Games-Howell [GH] corrected), while the difference between either CHR-N vs. HC group (*p* = 0.603 uncorrected) nor CHR-P vs. HC group contrasts was significant (*p* = 0.058 uncorrected). Although there were two outliers in the CHR-P group (see Supplementary Material), these did not significantly influence the results. In addition, none of the other LAUD metabolites (Figure 2) or ratio scores were significant (Glx/GABA *p* = 0.65, or Glu/GABA *p* = 0.85).

### MRS Data: *Post-hoc* CHR-P Follow-Up Subgroup Analyses

*Post-hoc* analyses of LAUD Glx concentrations in the CHR-P group included ANCOVAs with age as covariate for two different contrasts, one for clinical outcome (APS persistent vs. APS non-persistent) as well as for functional outcome (poor vs. good functional outcome).

A total of 17 CHR-P participants with LAUD cortex data were assigned to the APS-P group. For *n* = 20 CHR-Ps, their baseline-recorded APS symptoms remitted (non-persistent APS-NP group). These two groups did not significantly differ from each other in LAUD Glx concentrations (APS-P vs. APS-NP: *p* = 0.400).

In a second contrast, CHR-P participants with good functional outcome in 12 months (GAF scores ≥ 65, *n* = 23) were compared in their LAUD Glx concentrations to a group with poor functional outcome (GAF scores < 65, *n* = 32). These groups also did not differ from each other in their LAUD Glx concentrations (GAF high vs. GAF low: *p* = 0.286).

A total of 11 CHR-Ps developed a psychotic episode during the follow-up period (mean/standard deviation of 17.6 ± 8.3 months; 3 < 12 month, 2 at 12 months, 6 > 12 month). For 7 of these CHR-Ps, LAUD cortex MRS data was available. Compared to healthy controls, their Glx levels were not significantly affected (*p* = 0.84).

### MRS-Data Correlations

Left auditory cortex Glx data from CHR-N and CHR-P group were entered together into a linear regression analysis with backward selection of potential predictive variables, including AGE, GAF, GF-role, GF-social, SPI-A total severity, CAARMS total severity, CAARMS subscales, BACS composite scores and scores on six included tests (verbal memory, digit coding, token motor, verbal fluency, symbol coding, and Tower of London task). A significant model was found [*F*(3, 76) = 4.9, *p* = 0.003], including the predictors AGE (Beta = −0.321, *t* = −3.0, *p* = 0.003, VIF = 1.010), CAARMS Perceptual Abnormalities (Beta = −0.198, *t* = −1.8, *p* = 0.081, VIF = 1.134), and CAARMS Disorganized Speech (Beta = 0.226, *t* = 2.0, *p* = 0.047, VIF = 1.133) items, that showed the same trend for both groups (Figure 2). In summary, LAUD cortex Glx levels were lower in CHR-N/P individuals with higher age, higher severity of perceptual abnormality, but lower disorganized speech symptoms.

## Discussion

The current study examined alterations in E/I-balance parameters in auditory cortex to test the hypothesis that neural circuits during emerging psychosis are characterized by changes in GABA, Glx, or glutamate that would be consistent with previous evidence in ScZ (40–42). Moreover, we aimed to establish whether changes in these metabolites could also constitute a potential biomarker for clinical and functional outcomes in CHR-P participants.

The hypothesized reduction in Glx in CHR-P participants was only observed for the contrast involving CHR-Ns. Expected increases in GABA levels, however, were not found. Moreover, glutamate levels were unaffected in both left and right auditory cortex. A decrease in Glx without a change in glutamate levels could point to the reductions in Glx being more affected by glutamine than glutamate concentrations. Glutamine levels, however, could not be established due to too low SNR.

Furthermore, reductions in LAUD cortex Glx concentration in CHR-P individuals did not predict APS persistence or functional outcomes, in line with a recent study (56). Previous findings, however, reported that glutamate levels in CHR-Ps predicted the severity of positive symptoms at follow up, in particular abnormal thought content (57). Moreover, higher baseline striatal glutamate has been shown to predict transition to psychosis (32).

Our finding of lower left auditory cortex Glx in CHR-Ps is consistent with data in ScZ patients (41, 42, 58). Interestingly, decreased Glx concentrations have been found in particular in patients reporting more frequent and more severe auditory verbal hallucinations (58, 59). In the current study, higher Glx concentrations in CHR-Ps, however, were associated with higher disorganized speech ratings, rather than with perceptual abnormalities. It must be noted, however, that the perceptual abnormality score of the CAARMS combines aberrant perceptual experiences thus does not reflect subthreshold auditory hallucinations alone. As such, these findings support calls for more detailed assessments of hallucinations in CHR-P groups (60).

The lower Glx concentrations in CHR-Ps were exclusively found for the LAUD cortex, in line with the predominantly left lateralized effects reported in established ScZ (42). There are known structural and functional hemispheric differences in auditory cortex (61). Functionally, consensus has emerged that left auditory cortex is more sensitive to temporal cues than right auditory cortex (62) and that Glx levels differ between hemispheres (63, 64). Moreover, there is evidence for left, but not right, superior temporal cortex (STG) gray matter loss in FEP patients (65), and in CHR-P individuals (66), in particular those that later transition to psychosis (67). Recent meta-analyses on structural changes in both ScZ patients and CHR participants, however, suggested bilateral changes in cortical thickness (68, 69).

It should be noted that MRS data was collected on a 3T scanner, and sequences were optimized for GABA (*TE* = 68), which especially limits the ability to measure glutamate as a separable peak in the spectrum. Nonetheless, we believe our approach successfully estimated the contribution of this metabolite. Furthermore, the number of individuals with follow-up APS persistent and APS remitted status was relatively small.

In summary, the current findings failed to show robust deficits in GABA, glutamate and/or Glx in CHR-Ps, although a specific reduction was observed in Glx between CHR-P and CHR-N groups. Furthermore, across all CHR participants, stronger Glx reductions were linked to greater APS psychopathology, but did not correlate in CHR-Ps with clinical and/or functional outcome. These patterns suggest that auditory cortex Glx levels may potentially be useful in differentiating early-stage psychosis from non-psychotic disorders, but not in the prediction of functional or clinical outcomes.

## Data Availability Statement

The raw data supporting the conclusions of this article will be made available by the authors, without undue reservation.

## Ethics Statement

The studies involving human participants were reviewed and approved by the West of Scotland Research Ethics Service (14/WS/0099). Written informed consent to participate in this study was provided by the participants’ legal guardian/next of kin.

## Author Contributions

PU formulated research protocol and design, in collaboration with RG, JG, AG, SL, MS, and FS-L. TG-‘t-J was responsible for setting up MRS recordings, collecting data, conducting statistical analyses on the in JMRUI preprocessed data by SW, and writing of the report, together with PU. All authors have approved the final version.

## Conflict of Interest

The authors declare that the research was conducted in the absence of any commercial or financial relationships that could be construed as a potential conflict of interest.

## Publisher’s Note

All claims expressed in this article are solely those of the authors and do not necessarily represent those of their affiliated organizations, or those of the publisher, the editors and the reviewers. Any product that may be evaluated in this article, or claim that may be made by its manufacturer, is not guaranteed or endorsed by the publisher.
