# Supplementary material for: MR-Spectroscopy of GABA and Glutamate/Glutamine Concentrations in Auditory Cortex in Clinical High-Risk for Psychosis Individuals
Source: Front Psychiatry. 2022 Mar 29;13:859322. doi: 10.3389/fpsyt.2022.859322 (PMC9002006; doi:10.3389/fpsyt.2022.859322)
Supplement: Supplementary file 1 [file Data_Sheet_1.pdf]

MR-Spectroscopy of GABA and Glutamate/Glutamine concentrations in Auditory Cortex in Clinical High-Risk for Psychosis Individuals

Grent-‘t-Jong, Tineke, Ph.D., Ph.D., Ruchika Gajwani, Ph.D., Joachim Gross, Ph.D., Andrew I. Gumley, Ph.D., Stephen M. Lawrie M.D., Matthias Schwannauer, Ph.D., Frauke Schultze-Lutter, Ph.D., Stephen R. Williams, Ph.D.& Peter J. Uhlhaas, Ph.D.

MRS data: main group analyses – presence of outliers

Two CHR-P individuals had particularly low LAUD cortex Glx concentrations (z-values of -4.12 and -3.37, compared to the control group). The presence of outliers in the CHR-P group is not fully unexpected, given the clinical heterogeneity of psychopathology in this group, but poor data quality can also result in low values. Data quality for Glx of both individuals, however, was within the normal range (absolute CRLB for Glx: z-values of -0.98 and -1.29; LW water peak; z-values -1.24 and -1.29). The groups also had equal variances for Glx data (Levene’s test: $p = 0.302$). Shapiro-Wilk tests, however, revealed that the CHR-P group data was not normally distributed ($W = 0.953$, $p = 0.021$). We therefore repeated the analyses of LAUD cortex Glx concentrations, using a non-parametric approach. The results showed that there was still a main effect of GROUP (Kruskal-Wallis test: $H(2) = 6.2$, $p = 0.045$), as well as a significant post-hoc group difference between CHR-N and CHR-P groups (Mann-Whitney test: $U = 792$, $p = 0.033$). Moreover, as can be seen in Figure S1, taking out the two outliers did not significantly change these results (Mann-Whitney without outliers: $U = 752$, $p = 0.050$).

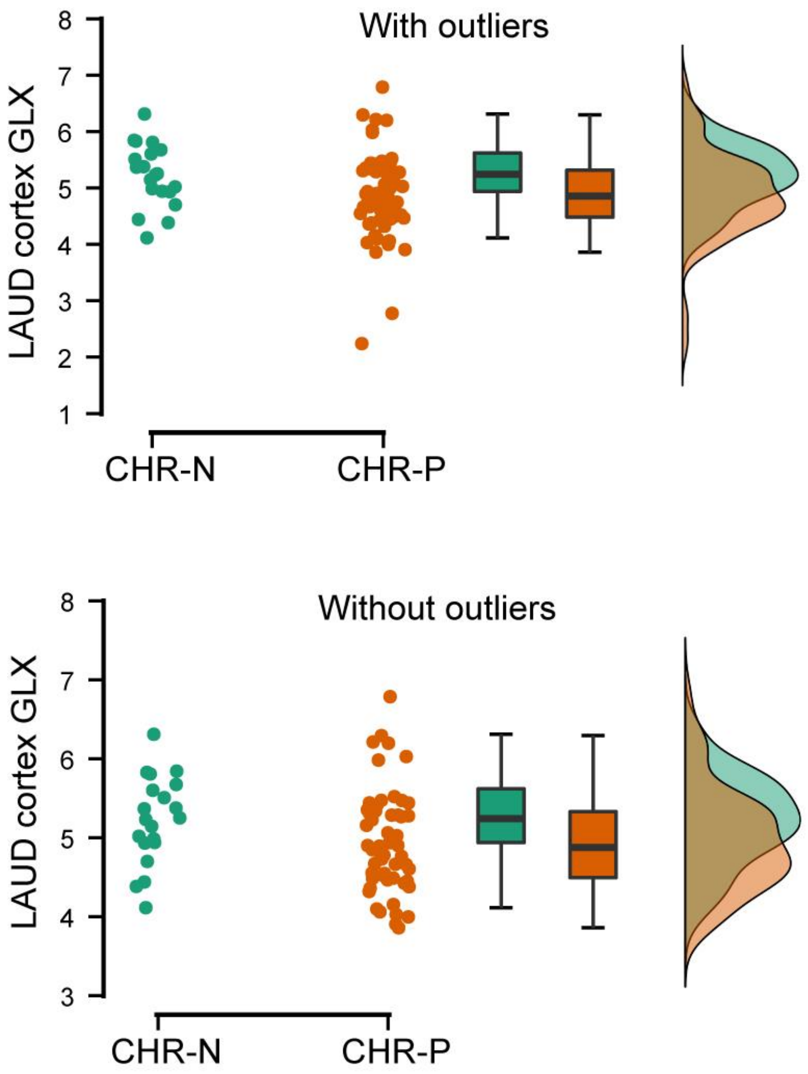

Figure S1. Left auditory cortex Glx concentration in CHR-N and CHR-P groups.

Distributions of Glx concentrations in left auditory cortex (referenced to water) for CHR-N and CHR-P groups, including raincloud plots, box and whisker plots as well as distribution overlay plots of both groups. The top-panel figure shows the data including two outlier CHR-P individuals, whereas these two outlier data points are not included in the lower panel figure, revealing minimal influence of the outliers. Abbreviations: Glx = Glutamate + Glutamine, CHR-N = Clinical-High-Risk negative: CHR-P = Clinical-High-Risk positive group.
